# *Zea mays* Taxilin Protein Negatively Regulates Opaque-2 Transcriptional Activity by Causing a Change in Its Sub-Cellular Distribution

**DOI:** 10.1371/journal.pone.0043822

**Published:** 2012-08-24

**Authors:** Nan Zhang, Zhenyi Qiao, Zheng Liang, Bing Mei, Zhengkai Xu, Rentao Song

**Affiliations:** Shanghai Key Laboratory of Bio-energy Crops, School of Life Sciences, Shanghai University, Shanghai, China; Nanjing Agricultural University, China

## Abstract

*Zea mays* (maize) Opaque-2 (ZmO2) protein is an important bZIP transcription factor that regulates the expression of major storage proteins (22-kD zeins) and other important genes during maize seed development. ZmO2 is subject to functional regulation through protein-protein interactions. To unveil the potential regulatory network associated with ZmO2, a protein-protein interaction study was carried out using the truncated version of ZmO2 (O2-2) as bait in a yeast two-hybrid screen with a maize seed cDNA library. A protein with homology to Taxilin was found to have stable interaction with ZmO2 in yeast and was designated as ZmTaxilin. Sequence analysis indicated that ZmTaxilin has a long coiled-coil domain containing three conserved zipper motifs. Each of the three zipper motifs is individually able to interact with ZmO2 in yeast. A GST pull-down assay demonstrated the interaction between GST-fused ZmTaxilin and ZmO2 extracted from developing maize seeds. Using onion epidermal cells as *in vivo* assay system, we found that ZmTaxilin could change the sub-cellular distribution of ZmO2. We also demonstrated that this change significantly repressed the transcriptional activity of ZmO2 on the 22-kD zein promoter. Our study suggests that a Taxilin-mediated change in sub-cellular distribution of ZmO2 may have important functional consequences for ZmO2 activity.

## Introduction

The *Zea mays* (maize) protein Opaque-2 (ZmO2) is a bZIP transcription factor that is primarily expressed in the sub-aleurone layers of maize endosperm [1], [2], [3], [4]. ZmO2 controls the synthesis of a major storage protein class in maize seed, the 22-kD zeins. In *opaque-2* mutants, the 22-kD zeins are reduced to 10% of the wild type level [2], [5], [6], [7], [8]. In addition, ZmO2 regulates the expression of 27-kD zeins, *b-32* (32-kD albumin), *ZLKRSDH* (lysine–ketoglutarate reductase/saccharopine dehydrogenase), *Ask1* (lysine-sensitive aspartate kinase) and *cyPPDK1* (cytoplasmic pyruvate orthophosphate dikinase) [2], [8], [9], [10], [11], [12], [13], [14]. ZmO2 is considered to be an important regulatory factor that controls the mass balance between protein and starch in maize seed [15].

In living cells, most physiological activities depend on protein-protein interactions. Because ZmO2 is an important transcription factor, it is subjected to multiple levels of regulation. ZmO2 is regulated upon synthesis, transport, function performance and degradation, most likely through interactions with other proteins. Previous studies revealed that a few proteins such as OHP1 (Opaque-2 heterodimerising protein 1), PBF (prolamin-box binding factor), GCN5 (general control of amino-acid synthesis protein 5) and ADA2 (transcriptional adaptor 2) can interact with ZmO2. OHP1 is a bZIP transcription factor and can bind to the ZmO2 target site as a homodimer or as a heterodimeric complex with ZmO2 [16]. PBF belongs to the Dof class of plant zinc-finger transcription factors and binds to the prolamin-box (P-box), which lies 20 bp upstream of the ZmO2 target site in the 22-kD zein gene promoter [17]. GCN5 and ADA2 are co-activators (or adaptors) that mediate the interaction between basic transcription factors and activators, which bind at specific sites. GCN5 and ADA2 form a protein complex with ZmO2, by direct or indirect interaction, to modulate the transcriptional activity of ZmO2 [18]. In addition, the DNA-binding activity of ZmO2 is regulated diurnally by a phosphorylation/dephosphorylation mechanism; consequentially it is likely that ZmO2 protein interacts with kinase (s) and phosphatase (s) [19], [20], [21]. Although there are some studies on interactions of ZmO2 with other proteins, the detailed modification and regulatory mechanisms of ZmO2 were not fully unveiled. To obtain additional information about the ZmO2 interaction network, a yeast two-hybrid screen was performed to identify proteins that interact with ZmO2.

In this study, a protein named ZmTaxilin was found to interact with ZmO2. The Taxilin homologues investigated in previous research are mainly from mammals [22], [23]. According to these reports, Taxilin genes have multiple functions. One function is to act as a binding partner of syntaxin family members and to take part in syntaxin-mediated vesicle trafficking [22], [23], [24], [25]. Another function is to interact with the nascent polypeptide-associated complex (NAC) and participate in transferring growing nascent polypeptide chains to appropriate co-translational factors [26]. Finally, a Taxilin homologous gene (FIAT) can repress transcriptional activity by dimerising with a bZIP factor (ATF4) to form inactive dimers that cannot bind the target site [27], [28], [29], [30], [31]. Our study finds that Taxilin interacts with the bZIP factor in the cytoplasm and alters the sub-cellular distribution of this transcription factor, which is a novel function of Taxilin.

The localisation and transport of proteins is highly selective and can be temporally regulated. For example, some transcription factors are maintained in an inactive state in the cytoplasm until a signal is received that promotes their translocation into the nucleus. These signals are usually proteins or chemical compounds from a particular transduction pathway [32], [33]. Because ZmTaxilin can change the localisation and repress the transcriptional activity of ZmO2, we speculate that ZmTaxilin might play a role in regulating ZmO2 activity in maize endosperm.

## Results

### Bait Vector Construction and Protein Interaction Screening

**Figure 1:**
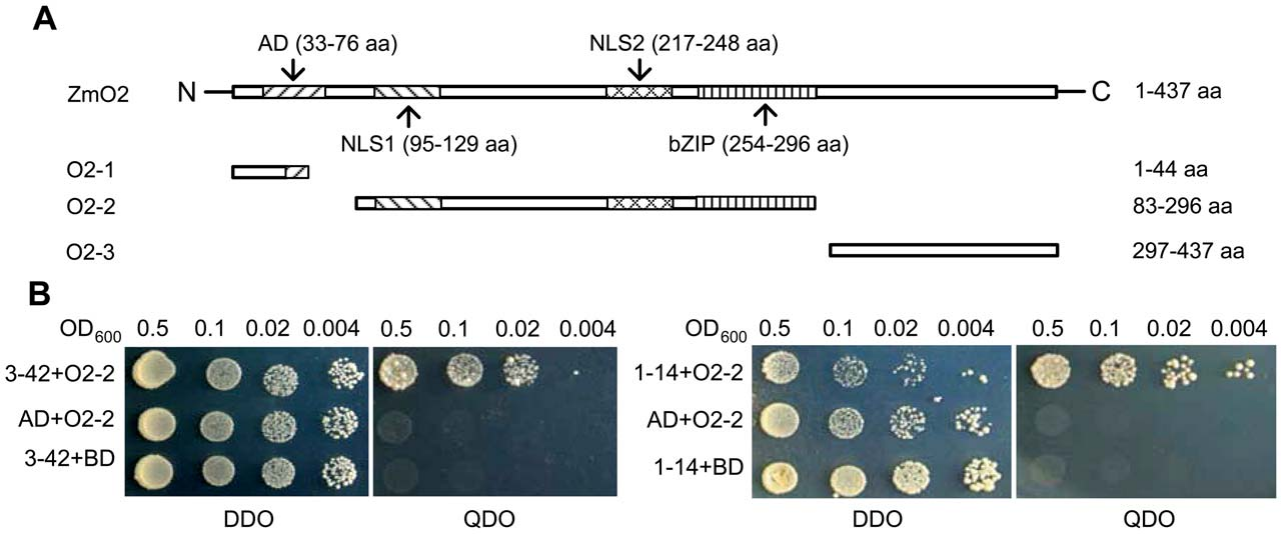
Baits construction and positive clones validation. (**A**) Structure of ZmO2 and three truncated versions of ZmO2 (O2-1, O2-2 and O2-3) [42]. AD: acidic activation domain; bZIP: basic leucine zipper domain; NLS: nuclear localisation signals. (**B**) Yeast two-hybrid analysis of the interaction between O2-2 and 3–42, and O2-2 and 1–14. Clones 3–42 and 1–14 are two of the five positive Taxilin clones (Table 1). AD is the GAL4 activation domain, and BD is the GAL4 DNA binding domain. 3–42+O2-2 (or 1–14+O2-2) represents the prey protein 3–42 (or 1–14) are co-expressed with the bait protein O2-2 in yeast cells. AD+O2-2 represents only AD domain without prey protein is co-expressed with the bait protein O2-2. 3–42+BD (or 1–14+BD) represents the prey protein 3–42 (or 1–14) is co-expressed with BD domain without bait protein. Yeast cells were cultured on DDO selective media (SD/−Leu/−Trp) and QDO (SD/−Ade/−His/−Leu/−Trp) in a dilution series indicated by the OD_600_ values displayed above the figure.

Because the ZmO2 protein has an activation domain, it resulted in a high background during the yeast two-hybrid screen when the full length ZmO2 was used as bait (data not shown). Therefore, three truncated versions of ZmO2, named O2-1, O2-2 and O2-3, were constructed (Fig. 1A). Bait vector pGBKT7-O2-1, pGBKT7-O2-2 and pGBKT7-O2-3 were co-transformed with pGADT7-Rec^m^ for the background test. The results show that O2-2 has the lowest background (data not shown). In addition, O2-2 contains the basic zipper domain, which often mediates protein-protein interactions in bZIP transcription factors. As a result, pGBKT7-O2-2 was selected as the bait vector for the yeast two-hybrid screen.

After screening approximately 10^6^ yeast transformants, 50 putative positive clones were obtained. Sequence analysis revealed that a gene homologous to Taxilin had 5 independent positive clones. All of the clones were confirmed to be in frame with the GAL4 protein, and they could be clustered into 3 different truncated versions when compared to the full-length cDNA of ZmTaxilin (GenBank Accession Number: BT063691) (Table 1). All clones were retransformed into AH109 with pGBKT7-O2-2 to confirm the interaction (Fig. 1B shows two of the clones named 1–14 and 3–42). The results indicate that the ZmTaxilin has stable interaction with O2-2 fragment in yeast.

**Table 1:** *ZmTaxilin* clones identified by yeast two-hybrid.

Clone number	Start position compared with full length cDNA	In frame or not
3–7	649 bp	Y
1–10	649 bp	Y
3–42	188 bp	Y
3–69	649 bp	Y
1–14	877 bp	Y

### Sequence Analysis of *ZmTaxilin* cDNA and Protein

**Figure 2:**
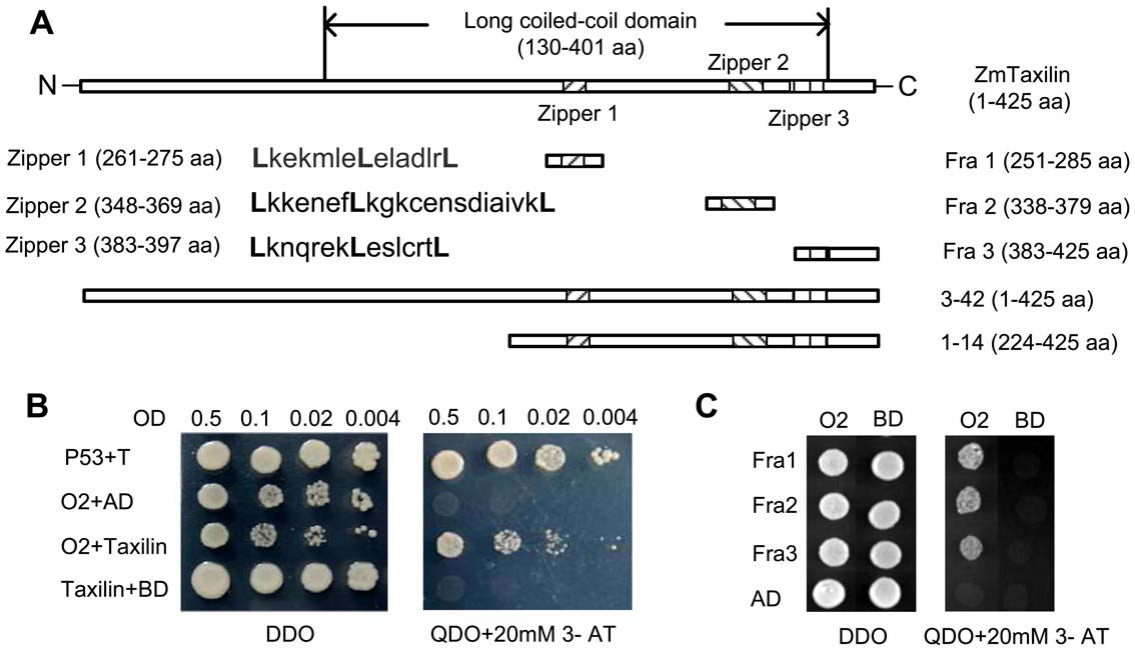
Yeast two-hybrid analysis of the interaction between ZmO2, full length ZmTaxilin and ZmTaxilin fragments. (**A**) Schematic representation of ZmTaxilin, Fra 1, Fra 2, Fra 3, 1–14 and 3–42. Fra 1, 2 and 3 are three fragments containing zippers 1, 2 and 3, respectively. 1–14 and 3–42 are two of the positive clones, and their corresponding proteins interact with O2-2 in yeast (Fig. 1B). (**B**) Full length ZmO2 interacts with full length ZmTaxilin in yeast cells. P53+T is positive control in which P53 (murine P53) is fused to BD and SV40 (large T antigen) is fused to AD [43]. O2+AD and Taxilin+BD are negative controls in which the BD-O2 fusion protein is co-expressed with AD or the AD-Taxilin fusion protein is co-expressed with BD. Yeast cells were cultured on selective media of DDO and QDO+20 mM 3-AT in a dilution. (**C**) Full length ZmO2 interacts with Fra 1, 2 and 3 in yeast cells. Prey protein Fra 1, 2 and 3 were fused to AD and were co-transformed with O2 and the BD fusion protein. AD+ZmO2, AD+BD, Fra1+BD, Fra2+BD and Fra3+BD are negative controls.

Blastx analysis indicated that one of the positive ZmTaxilin clones (3–42) contained a complete ORF and a 21 bp 5′ UTR. The ZmTaxilin gene contains a 1,278 bp predicted open reading frame, which encodes a polypeptide of 425 amino acids with a calculated molecular weight of 47.5 kDa. Conserved domain blast predicted that it has a long coiled-coil domain from 130 aa to 401 aa, which is shared by all Taxilin homologues (Fig. S1). Sequence analysis also revealed that ZmTaxilin has three conserved zipper motifs (Fig. 2A, Fig. S1). All zipper motifs contain a conserved leucine every seven amino acids. The leucine zipper domain is a structure that allows interaction with another leucine zipper by alignment of the leucine residues along the same face of the coiled-coil structure [34]. We speculated that these zipper motifs might mediate interactions with other proteins.

### Full Length ZmO2 can Interact with Full Length ZmTaxilin and Individual Zipper Motifs

As described above, the truncated ZmO2 (O2-2 fragment) could interact with the full length ZmTaxilin protein. To test if the complete ZmO2 protein could interact with ZmTaxilin, cDNAs with the complete *ZmTaxilin* and *ZmO2* ORFs were cloned into pGADT7-Rec^m^ and pGBKT7 respectively and co-transformed into yeast cells. The full length ZmO2 protein containing an activation domain causes auto-activation when used as the bait protein. Therefore, supplementation with 20 mM 3-AT (3-amino-1, 2, 4-triazole; a competitive inhibitor of the His3 protein) was used to control the background. On SD/−Leu/−Trp (Double Dropout, DDO) plates, all transformed cells grew well. On the SD/−Ade/−His/−Leu/−Trp (Quadruple Dropout, QDO)+20 mM 3-AT plates, yeast cells co-transformed with pGADT7-Rec^m^-Taxilin and pGBKT7-O2 grew well while cells transformed with either construct along with the responding empty vector could not grow (Fig. 2B). The results indicate that full length ZmO2 could interact with ZmTaxilin.

Amino acid sequence analysis of ZmTaxilin revealed that it has three zipper domains that are widely known to mediate protein-protein interactions (Fig. 2A). We speculated that the three zipper domains might mediate the protein-protein interaction of ZmO2 and ZmTaxilin. Three truncated fragments, which contain Zipper1, Zipper2 and Zipper3, were cloned into pGADT7-Rec^m^ (named pGADT7-Rec^m^-Fra1, 2 and 3, respectively). Yeast cells co-transformed with pGADT7-Rec^m^-Fra1/2/3 and pGBKT7-O2 were cultured on DDO and QDO+20 mM 3-AT. As shown in Fig. 2C, cells co-transformed with pGADT7-Rec^m^-Fra1/2/3 and pGBKT7-O2 grew well on QDO+20 mM 3-AT plates while cells transformed with any construct along with the responding empty vector did not grow. These data indicate that each of the three zippers is sufficient to interact with the full length ZmO2.

### Confirmation of the Interaction by a GST Pull-down Assay

A GST pull-down assay is one of the most common ways to demonstrate protein–protein interaction *in vitro*. Here, we performed a GST pull-down assay to determine whether ZmO2 and ZmTaxilin interact *in vitro*. Full length ZmTaxilin was fused to a GST tag and expressed in *E. coli*. The GST tag alone was expressed as a negative control. Native ZmO2 protein used in this assay was total protein extracted from immature maize seeds (15 DAP).

**Figure 3:**
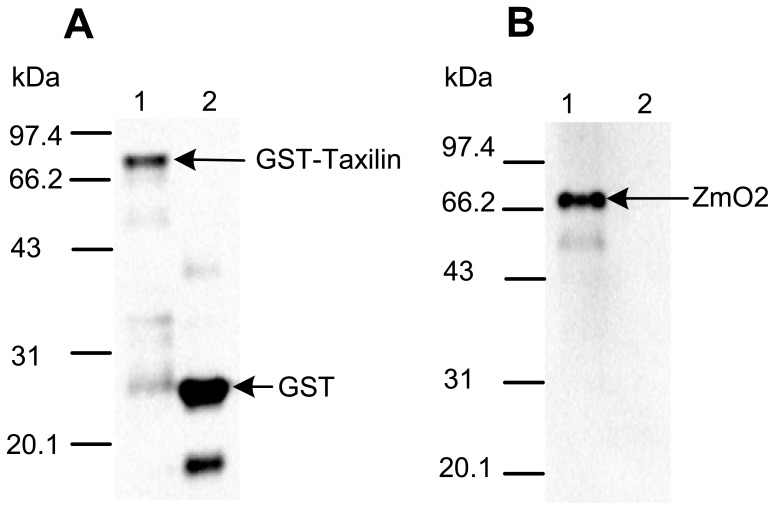
ZmTaxilin and ZmO2 interact in a GST pull-down assay. (**A**) Western blot detection of the GST pull-down sample with a GST antibody. The kernel sample was a pool of equal amounts of RNA from different developmental stages between 3 and 36 days after pollination (DAP). (**B**) Western blot detection of the GST pull-down sample with the ZmO2 antibody. Lane 1 in (**A**) and (**B**): *E. coli* lysate containing the GST-Taxilin protein and the maize seed protein containing ZmO2. Lane 2 in (**A**) and (**B**): *E. coli* lysate containing GST and maize seed protein containing ZmO2. The expected molecular weight of the GST-Taxilin fusion protein, the GST tag and ZmO2 are 75.397, 27.895 and 47.075 kDa, respectively. The apparent molecular weight of the ZmO2 protein was approximately 68–72 kDa.

The total protein from immature maize seeds (15 DAP) was incubated with beads bound to GST-Taxilin or GST alone. After washing with buffer, proteins retained by the beads were separated by SDS-PAGE. Western blot showed that both GST-Taxilin and the GST tag associated with the beads (Fig. 3A). The ZmO2 protein was efficiently pulled down by GST-Taxilin but not by the GST tag alone (Fig. 3B). These results indicate that ZmO2 and ZmTaxilin have a direct or indirect protein-protein interaction *in vitro*.

The cloned ZmO2 protein is 437 amino acids in length and has a predicted molecular weight of 47.075 kDa. However, the protein which expressed in maize endosperm migrates with an apparent molecular weight of approximately 68.0 kDa (Fig. 3B). In previous reports, the molecular weight of ZmO2 was approximately 68–72 kDa, and this is consistent with our results [19], [20], [21]. Nevertheless, the exact cause for the unusual migration of ZmO2 from maize endosperm is still unclear.

### Temporal and Spatial Expression of *ZmTaxilin*

**Figure 4:**
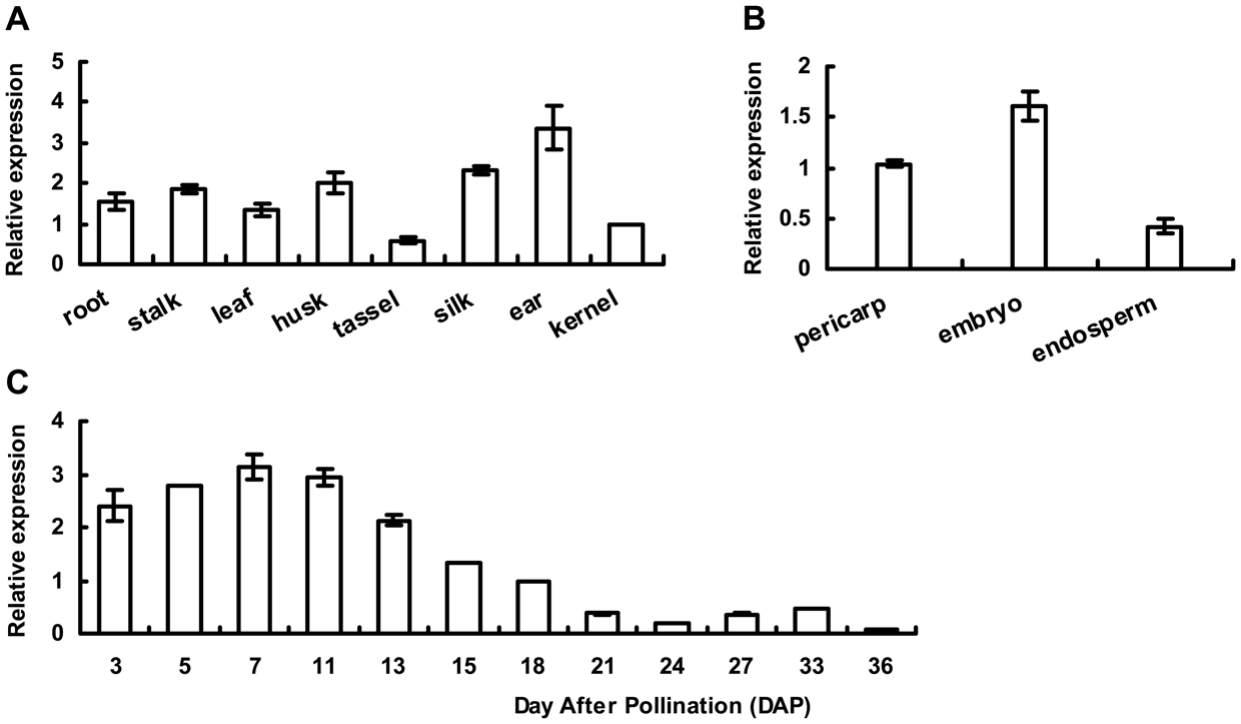
Temporal and spatial expression of *ZmTaxilin*. (**A, B**) Expression of *ZmTaxilin* in different tissues. (**C**) Expression of *ZmTaxilin* during maize seed development. For the temporal and spatial expression pattern, the quantity of *ZmTaxilin* in the in kernel and pericarp at 18 DAP was set to 1.

Real-time PCR was performed to investigate the expression pattern of *ZmTaxilin*. The results showed that *ZmTaxilin* is expressed in various tissues of maize, including the seed endosperm (Fig. 4A, B). During maize seed development, *ZmTaxilin* rose to a peak level approximately 7DAP and declined as the seed matured. Meanwhile, previous studies showed that *ZmO2* is mainly expressed in endosperm, and its transcript appears approximately 10 DAP and accumulate in the endosperm over a period of 20 days [3], [35]. These results indicate that the expression of *ZmTaxilin* and *ZmO2* has temporal and spatial overlap, which supports an interaction between ZmTaxilin and ZmO2 *in vivo*.

### ZmTaxilin Alters the Cellular Distribution of ZmO2

**Figure 5:**
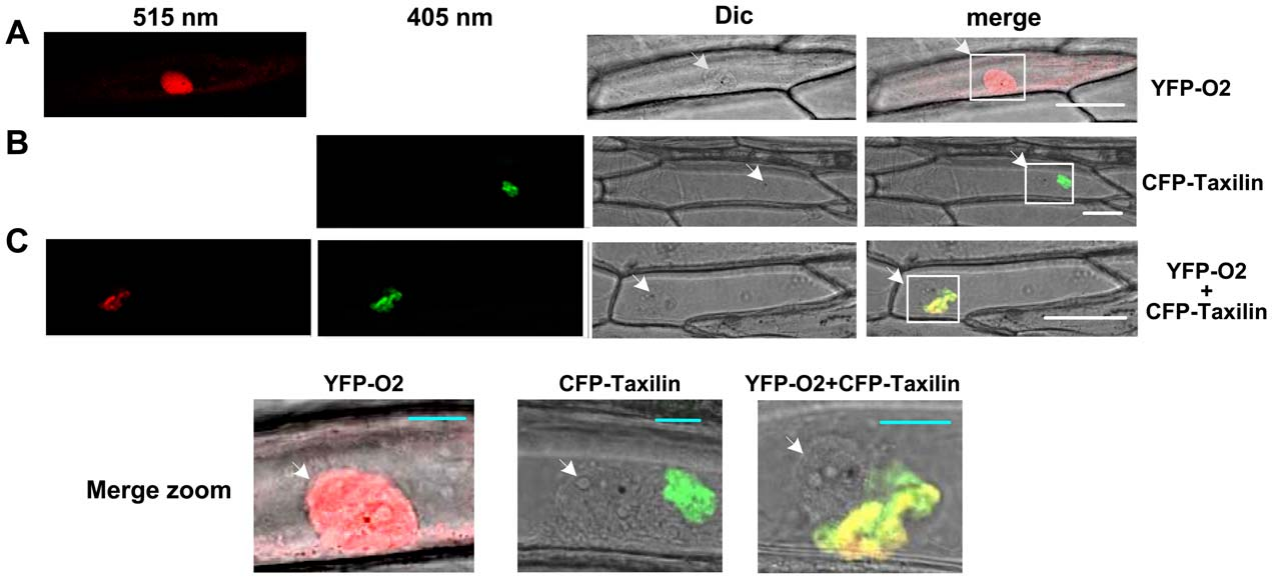
Co-localisation of ZmTaxilin and ZmO2 in onion epidermal cells. (**A**) YFP-O2 was transiently expressed in onion epidermal cells. (**B**) CFP-Taxilin was transiently expressed in onion epidermal cells. (**C**) YFP-O2 and CFP-Taxilin were co-expressed in onion cells. Differential interference contrast mode was used for bright-field microscopy. White Bars represent 100 µm and blue bars represent 20 µm. White arrows showed the localisation of the nuclei.

To investigate the sub-cellular localisation of ZmTaxilin and ZmO2, they were tagged with CFP and YFP to form CFP-Taxilin and YFP-O2. The fusion proteins were transiently expressed in onion epidermal cells individually or together. When *YFP-O2* was expressed alone, YFP-O2 localised predominantly to the nucleus and the cytoplasm (Fig. 5A, Fig. S2). When *CFP-Taxilin* was expressed alone, CFP-Taxilin was found to be clumped near the nucleus (Fig. 5B, Fig. S3). Interestingly, when *YFP-O2* and *CFP-Taxilin* were co-expressed in one onion epidermal cell, the location of the YFP-O2 protein changed and now like CFP-Taxilin, YFP-O2 was found near the nucleus (Fig. 5C, Fig. S4). The clumped localization of CFP-Taxilin might reflect its insolubility in onion epidermal cells. We also cannot specify the exact organelle where CFP-Taxilin is loacated in onion epidermal cells. However, YFP-O2 was completely repressed to be transported into nucleus when *YFP-O2* and *CFP-Taxilin* were co-expressed as the signal of YFP was only found near the nucleus. Taken together, the data indicate that the ZmTaxilin and ZmO2 co-localise *in vivo*, and ZmTaxilin can alter the sub-cellular distribution of ZmO2.

### ZmTaxilin Represses Transcriptional Activity of ZmO2

**Figure 6:**
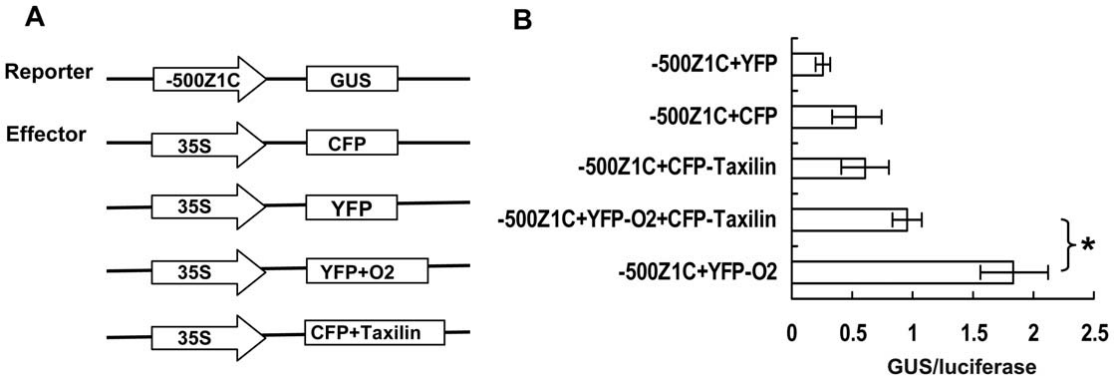
ZmTaxilin can repress the transcriptional activity of ZmO2. (**A**) Structure of effecter and reporters. (**B**) GUS/luciferase values of different reporter and effecter combinations. The values are the averages with SD of three independent experiments, after normalisation to the internal control. Statistical significance between YFP-O2 and YFP-O2+CFP-Taxilin was calculated using a two-tailed T-test. *p≤0.05.

To examine the effect of the interaction between ZmO2 and ZmTaxilin on the activity of ZmO2, a transient expression system containing reporter and effecter was developed in onion cells. The z1C promoter (500 bp upstream of the z1C gene *azs22.4* start codon) containing three ZmO2 binding sites was used to drive the GUS gene in the reporter plasmid (Fig. 6A). ZmTaxilin and ZmO2 were fused to CFP and YFP, respectively, and the fusion proteins were driven by the 35S promoter. In order to normalize the transformation efficiency an internal control vector 35S::LUC was constructed in which luciferase gene was driven by the 35S promoter. Reporter, effecter and internal control plasmids were co-transformed into onion cells by particle bombardment. The transcriptional activity of ZmO2 was determined by calculating the ratio of GUS/luciferase activities. The results indicate that YFP-O2 is able to induce transcription of the z1C promoter by 3–4 fold relative to the negative controls (CFP, YFP and CFP-Taxilin), demonstrating the effectiveness of this transcriptional assay system. However, when *YFP-O2* was co-expressed with *CFP-Taxilin*, the transcriptional activity on z1C promoter was reduced by 47% when compared to YFP-O2 alone (Fig. 6B). These results indicate that ZmTaxilin negatively regulates the transcriptional activity of ZmO2 when the two genes are co-expressed in onion cells.

## Discussion

ZmO2 is an important transcription factor that controls expression of a number of genes and plays an important role in starch/amino acid ratio balance during maize seed development [6], [8], [9], [10], [11], [12], [13], [14]. Intensive studies have revealed a variety of post-translational regulatory mechanisms for ZmO2, including interaction with co-activators or general transcription factors, and protein modification by phosphorylation/dephosphorylation [16], [17], [18], [19], [21]. In this study, we identified another possible regulatory mechanism for ZmO2 by regulation of its intracellular localisation.

We have identified a ZmTaxilin protein that interacts with ZmO2 supported by the following evidence. First, full length ZmO2 and ZmTaxilin can interact in yeast cells and *in vitro*. Second, the expression of *ZmO2* and *ZmTaxilin* overlap in time and space. ZmO2 transcripts appears approximately 10 DAP and remain constant in the following 20 days in maize seed [35]. Our Real-time PCR result showed that ZmTaxilin is expressed during the entire seed development process. Third, in an *in vivo* system (onion epidermal cells), ZmTaxilin not only co-localised with ZmO2, but also changed the localisation of ZmO2.

Our data suggest that the ZmTaxilin protein binds ZmO2 and sequesters ZmO2 in the cytoplasm, preventing ZmO2 from binding its target genes in the nucleus. In other words, the ZmTaxilin protein negatively modulates ZmO2 activity through the regulation of its intracellular localisation. Interestingly, previous studies have shown that other Taxilin proteins also associate with bZIP transcription factors and repress their transcriptional activity. For example, in early osteoblasts, the Taxilin protein (FIAT) dimerises with a bZIP transcription factor (ATF4) to form inactive dimmer that cannot bind DNA; in this case, the proteins interacted in the nucleus [30]. However, our observations suggest a different mechanism, which is to sequester the bZIP factor in cytoplasm to inhibit its activity. Furthermore, the interaction domains of ATF4 and FIAT are different from ZmO2 and ZmTaxilin. ATF4 only interacts with the second zipper of FIAT, but ZmO2 could interact with any of the three zippers of ZmTaxilin [29]. Taken together, our results suggest that Taxilin proteins regulate bZIP factors by a different regulatory mechanism.

The interaction between ZmO2 and ZmTaxilin has been validated in heterologous cells (yeast and onion epidermal cells) and *in vitro*. Although expression of ZmTaxilin and ZmO2 has overlap in time and space, transcript level does not always correlate with protein level. An antibody to ZmTaxilin will be made to detect the protein abundance during endosperm development. The *in vivo* localisation of the two proteins in maize seeds will be detected by immunolocalization and their ability to interact will be tested by co-immunoprecipitation. This work will further reveal the mechanism and biological significance of ZmO2 and ZmTaxilin interaction. Because ZmTaxilin behaves as a negative regulator of ZmO2 activity, our study provides a potential regulatory target to modulate the expression of major zein class-genes and other genes involved in seed-quality improvement in maize.

## Materials and Methods

### Plant Growth Conditions

Maize inbred line W22 was grown during the summer in a greenhouse (30°C day/20°C night, and 12 hours of light). After self-pollination, kernels were harvested between 3 and 36 days after pollination (DAP), immediately frozen in liquid nitrogen and stored at −80°C for RNA and protein extraction. Root, stalk, the third leaf, tassel, husk, silk and ear tissues were harvested at the V12 stage. Endosperm, pericarp and embryo were all dissected from maize seeds at 15 DAP. Tissue samples were collected from at least three individual plants for each development stage.

### Construction of a Yeast Two-hybrid Library

Total RNA was extracted from kernels harvested at different days, and then mRNA was enriched using PolyA Tract^R^ mRNA Isolation System III (Promega). Full-length cDNA synthesis was performed using Creator™ SMART™ cDNA library construction kit (Clontech). The full-length cDNA abundance was normalised with TRIMMER-DIRECT cDNA Normalisation Kit (Evrogen). The yeast two-hybrid vector pGADT7-Rec was purchased from Clontech. The pGADT7-Rec vector was converted into pGADT7-Rec^m^ in which one of the *Sfi*Ι sites at 1974 bp was mutated. The cDNA fragments were inserted into pGADT7-Rec^m^ between the two remaining *Sfi*Ι sites. The ligated product was transformed into ELECTROMAX DH10B COMP CELLS (Invitrogen) by electroporation, and the number of transformants was counted.

The final yeast two-hybrid library contained approximately 1.0×10^6^ clones. All of the clones were spread on plates. After culturing, the clones were scraped off the plates, and the plasmids were extracted.

### Bait Construction, Yeast Two-hybrid Screening and Analysis of Positive Clones

Full length *ZmO2* and three fragments from different part of *ZmO2* were inserted into the *Nde*Ι and *Sma*Ι sites in pGBKT7 to form pGBKT7-O2, pGBKT7-O2-1, pGBKT7-O2-2 and pGBKT7-O2-3 (Fig. 1A). The four bait vectors were co-transformed into yeast cell line AH109 with pGADT7-Rec^m^ using LiAc method. The transformants were spread on QDO (SD/−Ade/−His/−Leu/−Trp) plates containing 3-AT (3-amino-1, 2, 4-triazole; a competitive inhibitor of the His3 protein), and incubated at 30°C for 4–6 days for the background test. The transformants were simultaneously spread on DDO (SD/−Leu/−Trp) plates for transformation efficiency calculation. The vector pGBKT7-O2-2 was selected as the bait to screen for proteins that interact with ZmO2 and was transformed into AH109 yeast cells. The yeast cells containing pGBKT7-O2-2 were made into competent cells and transformed with yeast two-hybrid plasmids containing a maize seed cDNA library. Transformants were spread on QDO. The plates were incubated at 30°C for 5 days to allow positive clones to grow. Plasmids from all the positive clones were extracted and amplified in *E. coli*. All of the clones were sequenced, and all of the sequences were analysed by GenBank Blastx analysis [36]. The sequences that aligned to the same gene were grouped together.

### Yeast Two-hybrid Analysis of the Interaction between Different Bait and Prey

All of the prey proteins (ZmTaxilin, 3–42, 1–14, Fra 1, Fra 2 and Fra 3) used in this study were inserted into pGADT7-Rec^m^ in-frame with the GAL 4 activation domain (AD). pGBKT7-P53 and pGADT-RecT were used as positive controls where P53 (murine P53) fused to BD and SV40 (large T antigen) fused to AD. pGBKT7-P53 and pGADT-RecT are both from Matchmaker™ Library Construction & Screening Kits (Clontech).

Both vectors expressing prey and bait proteins were co-transformed into yeast AH109, and the cells were spread on DDO (SD/−Leu/−Trp) plates. After colonies were observed on plates, they were then incubated in liquid DDO. The cells were diluted in series according their OD_600_ values and cultured on DDO and QDO plates for further analysis. When the ZmO2 bait protein was full length, 20 mM 3-AT (3-amino-1, 2, 4-triazole; a competitive inhibitor of the His3 protein) was added to QDO to control the background.

### Protein Expression, Extraction, ZmO2 Polyclonal Antibody Preparation and GST Pull-down Assay

Full length *ZmTaxilin* was cloned into pGEX-4t-1 to express the GST-Taxilin fusion protein. The expression vectors were transformed into Rosetta (DE3). The GST-Taxilin and GST tag were induced by culturing *E. coli* at 30°C with 0.5 mM IPTG at 250 rpm for 5 hours. The cells were spun down and the pellet was resuspended in TBS buffer (25 mM Tris-HCl, 0.15 M NaCl, pH 7.2). The cells were lysed by ultrasonication, and the protein quantity was determined by the Bradford method.

The 15 DAP seeds of W22 were ground in liquid nitrogen and extracted with Maize Seed Extraction Buffer (150 mM NaCl, 50 mM Tris-HCl, pH 7.4–7.5, 2.5 mM EDTA, pH 7.4–7.5, 0.2% NP-40, 2.5 mM PMSF, 1% cocktail (Sigma)). The protein quantity was determined by the Bradford method.

The full length ORF of ZmO2 (GenBank Accession Number: BT016530) was inserted into the pet-32a vector for expressing the His-O2 fusion protein. The expression vectors were transformed into Rosetta (DE3) for protein expression. The His-O2 protein was induced at 30°C with 1 mM IPTG at 250 rpm for 5 hours. The His-O2 fusion protein was purified with the 1 ml HisTrap™ FF crude column (GE Healthcare Bio-Sciences). The purified protein was injected into rabbits to produce antibodies to His-O2. The ZmO2 antibodies were further purified by acetone powder that prepared from Rosetta cells expressing the His Tag.

Approximately 20 µg *E. coli* cell lysate containing GST-Taxilin protein was incubated with 25 µl Immobilised Glutathione beads (Pierce) for 1 hour at 4°C by gently shaking. Then, the beads were washed 3 times with 400 µl TBS and another 3 times with 400 µl Maize Seed Extraction Buffer. Next, the beads were incubated with 20 µg maize seed protein at 4°C for 2 hours. The beads were washed 5 times with TBS buffer, and the protein was eluted in 50 µl Glutathione Elution Buffer (100 mM Glutathione). The bound proteins were analysed by SDS-PAGE and western blot with GST antibody (Abcam) and ZmO2 antibody, respectively.

### Quantitative Real-time PCR Analysis of ZmTaxilin

Total RNA was extracted using Trizol Reagent (TIANGEN) and was treated by RNase-free DNaseΙ (Takara). All purified RNA samples were quantified using a Spectrophotometer and were used to synthesize cDNA with ReverTra Ace reverse transcriptase (Toyobo).

A pair of primers named ZmTaxilin (+) (5′-TCAACCTACCGTCCGTCTCAG-3′) and ZmTaxilin (−) (5′-GTAGGAATGCACCACTAGGCTCT-3′) were designed to amplify the 3′-UTR of *ZmTaxilin* (GenBank Accession Number: BT063691). Another pair of primers ZmUBQ (+) (5′-CTGGTGCCCTCTCCATATGG-3′) and ZmUBQ (−) (5′-CAACACTGACACGACTCATGACA-3′) were used to amplify the internal control gene *ZmUBQ* (GenBank Accession Number: BT018032).

Real-time PCR was carried out with a DNA Engine Option2 System (MJ Research) using SYBR Green Mix (Takara). The Ct (2^−ΔΔCt^) method was used to analyse the comparative expression of *ZmTaxilin*. In the temporal and spatial expression pattern analysis, the quantity of *ZmTaxilin* at 18DAP, in kernel and in pericarp was defined as 1. All of the data were repeated on three individual plants.

### Localisation and Co-localisation of ZmO2 and ZmTaxilin

The *ZmTaxilin* and *ZmO2* cDNA fragments were cloned into the gateway entry vector pENTR/D-TOPO (Invitrogen). The entry vectors were then recombined with pK7YWG2 and pB7CWG2 to generate YFP-O2 and CFP-Taxilin fusions proteins driven by 35S promoter [37]. Living onion (*Allium cepa*) epidermal cells were cultured on 1/2×MS media and bombarded with 1 µg superhelical plasmid DNA with the Biolistic PDS-1000/He Gene Gun System (Bio-Rad). Bombarded epidermal cells were incubated for 16 to 24 hours at 22°C in continuous darkness. Confocal imaging was performed using a ZEISS LSM 710 confocal microscope. The CFP protein was excited with an argon laser at 405 nm, and fluorescence emission was detected between 440 and 485 nm. To visualise the YFP protein, an excitation wavelength of 515 nm was used, and the emission window was set between 520 and 550 nm.

### Construction of Effecter and Reporter Plasmids and Co-expression in Onion Epidermal Cells

In a previous report, the *azs22.4* gene (GenBank Accession Number: Q9AR72) had the highest expression level among the 22-kD alpha zein gene family [38]. Its promoter has three ZmO2 binding sites [39]. Its promoter sequence (from −500 bp to ATG) was cloned and used to drive expression of *GUS* gene in the reporter plasmid. The effecter plasmids containing CFP-Taxilin and YFP-O2 protein were the same plasmids used for localisation. Empty pK7YWG2 and pB7CWG2 plasmids, which expressed YFP and CFP, respectively, were as the negative controls. To normalize the transformation efficiency an internal control vector named 35S::LUC was constructed in which luciferase gene was driven by the 35S promoter.

Based on previous reports, we determined that a range of plasmid DNA concentrations, from 100 ng to 10 µg, might be ideal for transformation of onion epidermal cells [40], [41]. Then, we carried out several preliminary experiments to choose the appropriate amount of plasmid DNA. We found that when we used 1 µg of plasmid, the GUS activity was in a suitable range for measurement. Consequentially, 1 µg of plasmid DNA was used to transform onion epidermal cells.

Effecter plasmids (1 µg) were co-transfected with reporter plasmids (1 µg) at a ratio of 1∶1. The efficiency of transfection was normalizing by adding 1 µg of 35S::LUC reporter plasmids. Onion epidermal cells were prepared the day before and bombarded with gold particles. The particles were coated with a mixture of plasmids encoding the reporter, effecter and internal control.

After one day of culturing at 25°C in darkness, an approximately 2×2 cm^2^ piece of the onion epidermal cells was ground in liquid nitrogen, and the protein was extracted with the GUS cell culture lysis reagent (100 mM Potassium phosphate pH 7.8, 1 mM EDTA pH 7.8, 1% Triton X-100, 10% Glycerol, 7 mM β-Mercaptoethanol). Twenty microliters of supernatant was added to 180 µl pre-warmed 2 mM 4-MUG. At 0, 10, 30 and 60 min, 10 µl of the reaction mixture was added to 190 µl 0.2 M sodium carbonate solution. The samples were excited at 365 nm and the fluorescence was read at 455 nm at different times with a microplate reader (TECAN Infinite M200). For the luciferase assays, 10 µl of supernatant was diluted in 190 µl of the luciferase assay reagent (20 mM Tricine pH 7.8, 5 mM MgCl_2_, 0.1 M EDTA, 3.3 mM DTT, 0.27 mM Coenzyme A, 500 µM Luciferin, 500 µM ATP). Luciferase activity was detected with a microplate reader using an excitation of 488 nm and an emission of 533 nm. All transfection assays were performed in triplicate.

## Supporting Information

Figure S1 **Alignment of ZmTaxilin protein with other Taxilin homologous proteins.** Yellow, green and blue background means the sequence was identical, weakly similar and conservative respectively. There is an obvious coiled-coil domain at 200–450 aa. In the coiled-coil domain there are three conserved zipper domain which have a conserved leucine acid every seven amino acid.
(PDF)

Figure S2 **Localization of YFP-O2 in onion epidermal cells.** Onion epidermal cells were bombarded with YFP-O2. Red represents YFP fluorescence. Differential interference contrast (DIC) was used in light mode.
(PDF)

Figure S3 **Localization of CFP-Taxilin in onion epidermal cells.** Onion epidermal cells were bombarded with CFP-Taxilin. Green represents CFP fluorescence. Differential interference contrast (DIC) was used in light mode.
(PDF)

Figure S4 **Co-localization of CFP-Taxilin and YFP-O2 in onion epidermal cells.** Red represents YFP fluorescence and green represents CFP fluorescence. Differential interference contrast (DIC) was used in light mode.
(PDF)
