# Supplementary material for: Zea mays Taxilin Protein Negatively Regulates Opaque-2 Transcriptional Activity by Causing a Change in Its Sub-Cellular Distribution
Source: PLoS One. 2012 Aug 24;7(8):e43822. doi: 10.1371/journal.pone.0043822 (PMC3427180; doi:10.1371/journal.pone.0043822)
Supplement: Figure S1 — Alignment of ZmTaxilin protein with other Taxilin homologous proteins. Yellow, green and blue background means the sequence was identical, weakly similar and conservative respectively. There is an obvious coiled-coil domain at 200–450 aa. In the coiled-coil domain there are three conserved zipper domain which have a conserved leucine acid every seven amino acid. (PDF) [file pone.0043822.s001.pdf]

```
                                         1                                                50
                 ACN28388 zea mays   (1) MEGSPVARLPEANSLPDGFVPDSESSDTDAAPPSSAPIVDDAIDSDSPDA
     XP_002468045 Sorghum bicolor    (1) MEGSPVTRLPEANTLPDGFVPDVESSGTDAASPSSAPIAADALDSDIPAA
      XP_002276892 Vitis vinifera    (1) MENTAANQLPEVDSLPDGFVESSAEPVAPPTPTLDKES----DYKDSNFS
 XP_002316353 Populus trichocarpa    (1) MENRVANQLPEVDSLPDGFVDSSMEPVAPQTPTSEQEKPVSDCKEDSIGK
    XP_002529722 Ricinus communis    (1) MDNVDANQLPEVDSLPDGFVDGSIESLAPPTPPFEQEKPLSVRNKDSISE
                        Consensus    (1) MENS ANQLPEVDSLPDGFVD S ESVAP TPPSE E        DS  A
                                         51                                              100
                 ACN28388 zea mays  (51) TNPGGEETLSDPSLPASTAEDASSAAAAEALD------------------
     XP_002468045 Sorghum bicolor   (51) TNPGGEETLSDLSLPASAAEDASSAAAAEALG------------------
      XP_002276892 Vitis vinifera   (47) TTEP-SNDLENESLELLDSSVDRVEKTQKPR-------------------
 XP_002316353 Populus trichocarpa   (51) VDHV-SEALQELAANESQTSQNGTEKPEKMRNFPVPLSDIDGCDASVHLV
    XP_002529722 Ricinus communis   (51) IVHP-TGGLPEFSANESQTSQESNEKVGNMR-----------TLFDASVL
                        Consensus   (51) T    SE L D SL  S TS  SSEK A  R
                                         101                                             150
                 ACN28388 zea mays  (83) ------------------TLCLDVVADPERALGEHGPTTAARGEGSLKEN
     XP_002468045 Sorghum bicolor   (83) ------------------TLSLAAAAEPERALGEHGPTGAARGEETLKEN
      XP_002276892 Vitis vinifera   (77) ------------------TFPVPLSEKDGFDSSGESPKVTNKGCSEPGEA
 XP_002316353 Populus trichocarpa  (100) VEPDQGAWQEEGTLTKPVLDDLVPEASVGVSRCSEVIEVQ-GSSQSSDRS
    XP_002529722 Ricinus communis   (89) IVDHAHVEQVEGTATILDSANLVSNTSIEVSGCSELNEQVSGNCQSSDRS
                        Consensus  (101)                   T L   A  E A   E P   ARG  S  ES
                                         151                                             200
                 ACN28388 zea mays (115) R---------ASEQVGAPSDQKVVKGS----GEPKRKVIKHSKLEKDKEL
     XP_002468045 Sorghum bicolor  (115) C---------ASEQVGAPTAQKVVKGS----GEPKRKVGKRSKLERDREL
      XP_002276892 Vitis vinifera  (109) TEGGSDAPTTHLKDVSSSESIEHLKNRKLETSEVKRKSAKRT-FKSEKEF
 XP_002316353 Populus trichocarpa  (149) TQGGLDSQATGVKEISSSESTELQKGR-KEATETKRKNAKRT-FKSEKEF
    XP_002529722 Ricinus communis  (139) VQGGSSTYATDVKEMSSSESLQSQKGRKLETPEAKRKNPKRS-FKSEKEF
                        Consensus  (151)   GG      TAVKEVSSSES  VVKGR  E  E KRK AKRS FKSEKEF
                                         201                                             250
                 ACN28388 zea mays (152) FQLAQQYHKVVAERDQAIAVKDRLESLCREFQRQNKMLKEECQRVSTEGQ
     XP_002468045 Sorghum bicolor  (152) FELAQQYHKVVAERDAAILVKEKLESLCREFQRQNKTLKEECQRVSTEGQ
      XP_002276892 Vitis vinifera  (158) LEFSLKYQQVIAERDTAIAVRDKLESLCRELQRQNKLLMDECKRVSAEGQ
 XP_002316353 Populus trichocarpa  (197) LEFTLKYQQVLTERDAAIVVRDKLESLCRELQRQNKMLMDECKRVSTEGQ
    XP_002529722 Ricinus communis  (188) LEFTLKYQQVLAERDAAIAMRNKLESLCRELQRQNKMLMEECKRVSTEGQ
                        Consensus  (201) LEFTLKYQQVLAERDAAIAVRDKLESLCRELQRQNKMLMEECKRVSTEGQ
                                         251                                             300
                 ACN28388 zea mays (202) NMRTELSEKFDHAIKGVSAKLEEQRVERISQLEENNTLRSKLKDLADQYN
     XP_002468045 Sorghum bicolor  (202) NMRMALSEKFDNAIKGVSAKLEEQRVLSIAQLEENNMLRSKLNDLADQYS
      XP_002276892 Vitis vinifera  (208) TLRLDLSTKFQDAIKDVSSKLEEQKDECLSQLKENEMLRNKLKQLADQYT
 XP_002316353 Populus trichocarpa  (247) HLRLDLSTKFQDAIKDVSNRLEEQKEESLTQLKENEMLRKKLKEFADQYA
    XP_002529722 Ricinus communis  (238) TLRLDLSTKFQDAIKDVSIRLEEQKEECFSQLKENEMLRTKLKQLADQYA
                        Consensus  (251)  LRLDLSTKFQDAIKDVSAKLEEQKEE ISQLKENEMLRSKLKDLADQYA
                                         301                                             350
                 ACN28388 zea mays (252) ITQQKYAHQLKEKMLELELADLRLQQHQEKAAQEHTQMQLYAEQVSQLMT
     XP_002468045 Sorghum bicolor  (252) ITQQKYAHQLKEKTLELELADLRLQQNQEKAAQEHTQMQLYAEQVAQLMT
      XP_002276892 Vitis vinifera  (258) LSEQQFAQKLKQKTLELQLADLKNQQHEEKLIQEQSQMKLYAEQVSQLLT
 XP_002316353 Populus trichocarpa  (297) ISEQQNAQKLKQKTLELQIADLKIKQHEEKLVQEQSQMKIYAEQVSQLLA
    XP_002529722 Ricinus communis  (288) VSEQQHAQKLKQKSLELQIAELKIKQHEEKLVHEQSQMKVYAEQVSQLLA
                        Consensus  (301) ISEQQYAQKLKQKTLELQLADLKIQQHEEKLVQEQSQMKLYAEQVSQLLT
                                                    Zipper1
                                         351                                   400
                 ACN28388 zea mays (302) TEKNLRLQLASDGERFQHFQDALSKSNEVFETYKQEMEKMISVIKNLKKE
     XP_002468045 Sorghum bicolor  (302) TEKNLRLQLASDGDRFQHFQDALSKSNEVFETYKQEMEKMISVIKNLKKE
      XP_002276892 Vitis vinifera  (308) TEKSLRLQLTADGEKFQQFQEALLKSNEVFETFKQEIEKMAKSIKELKKE
 XP_002316353 Populus trichocarpa  (347) TEKTLRLQLTADGDKFQQFQEALVKSNEVFETFKQEIDKMAKSIKELKKE
    XP_002529722 Ricinus communis  (338) TEKNLRMKLTADGEKFQQFQDALLKSNEVFETFKQEIEKRTKSIKKLKKE
                        Consensus  (351) TEKNLRLQLTADGEKFQQFQDALLKSNEVFETFKQEIEKM KSIK LKKE
```

```
                                               401                                              450
ACN28388 zea mays                  (352) NEFLKGKCENSDIAIVKLIEERELTKKQIEKLKNQREKLESLCRTLQAER
XP_002468045 Sorghum bicolor       (352) NEFLKGKCENSDFALVKLIEERELTKKQLEKLKNQKEKLESLCRSLQAER
XP_002276892 Vitis vinifera        (358) NIFLKSKSEKSDVTLIELVEERERLKKQLEKMKNQKEKLESLCRSLQAER
XP_002316353 Populus trichocarpa   (397) NTFLKSKSEKSDVMLIELVDEREKLKKQLDKTRNQKEKLESLCRSLQAER
XP_002529722 Ricinus communis      (388) NVFLKSKTEKSDVTLIELVEERERLKKQLEKTKNQKEKLESLCRSLQAER
Consensus                          (401) N FLKSKSEKSDV LIELVEERERLKKQLEKLKNQKEKLESLCRSLQAER
                                            Zipper2                     Zipper3

                                         451                      477
ACN28388 zea mays                  (402) KQGPS-ASIPDAPSSQ-EVSATSQES-
XP_002468045 Sorghum bicolor       (402) KQGPS-ASIPDAPSSQEDVSATSQES-
XP_002276892 Vitis vinifera        (408) RQNSIGSNASD----------------
XP_002316353 Populus trichocarpa   (447) KQNKTGSNSSDSVPA------------
XP_002529722 Ricinus communis      (438) KQNSNGGNTPDTVRNDH----------
Consensus                          (451) KQN SGAN PDA  S
```

Figure S1. Alignment of ZmTaxilin protein with other Taxilin homologous proteins. Yellow, green and blue background means the sequence was identical, weakly similar and conservative respectively. There is an obvious coiled-coil domain at 200-450aa. In the coiled-coil domain there are three conserved zipper domain which have a conserved leucine acid every seven amino acid.
