# Supplementary material for: Zea mays Taxilin Protein Negatively Regulates Opaque-2 Transcriptional Activity by Causing a Change in Its Sub-Cellular Distribution
Source: PLoS One. 2012 Aug 24;7(8):e43822. doi: 10.1371/journal.pone.0043822 (PMC3427180; doi:10.1371/journal.pone.0043822)
Supplement: Figure S3 — Localization of CFP-Taxilin in onion epidermal cells. Onion epidermal cells were bombarded with CFP-Taxilin. Green represents CFP fluorescence. Differential interference contrast (DIC) was used in light mode. (PDF) [file pone.0043822.s003.pdf]

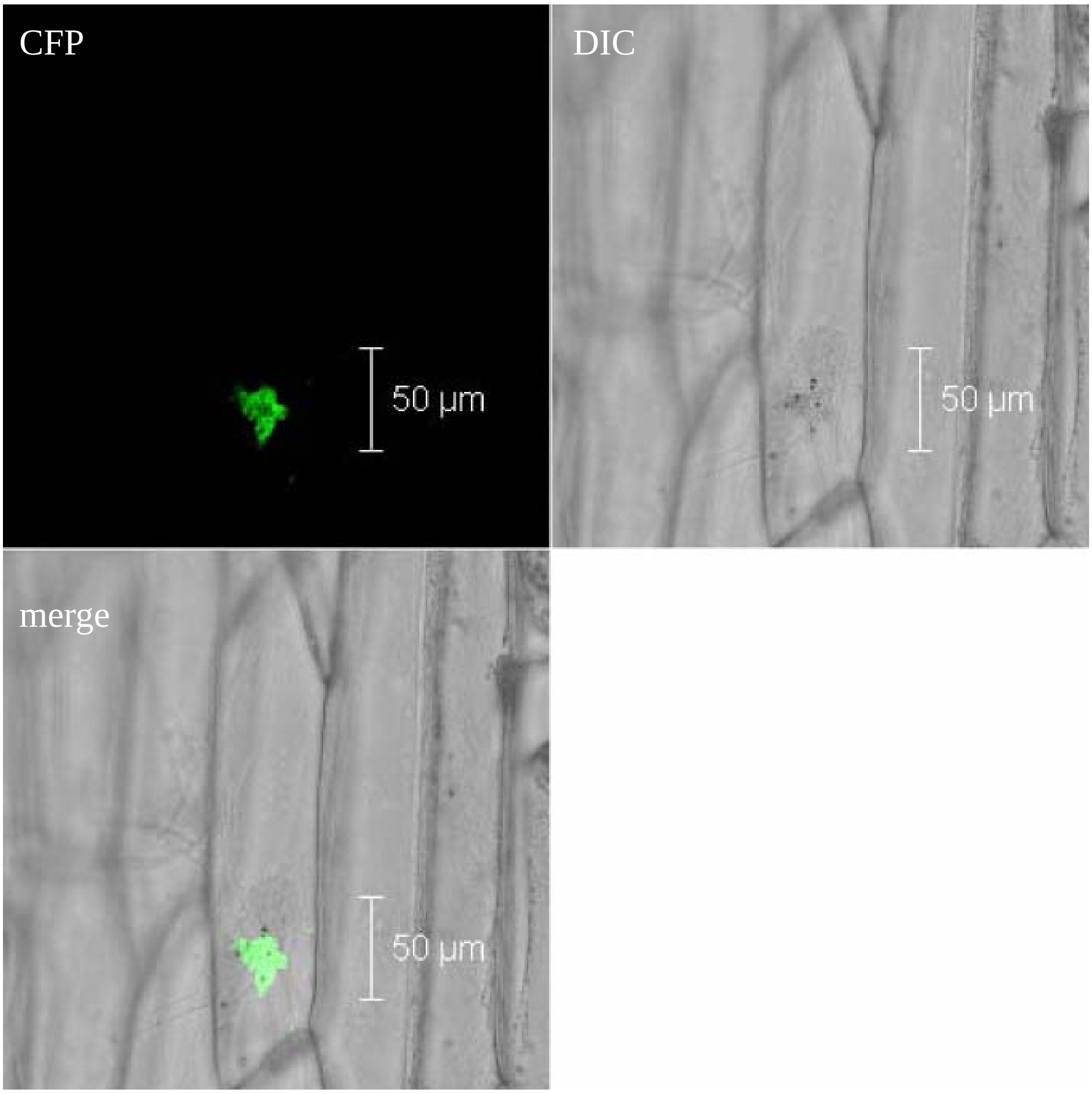
CFP
50 µm
DIC
50 µm
merge
50 µm

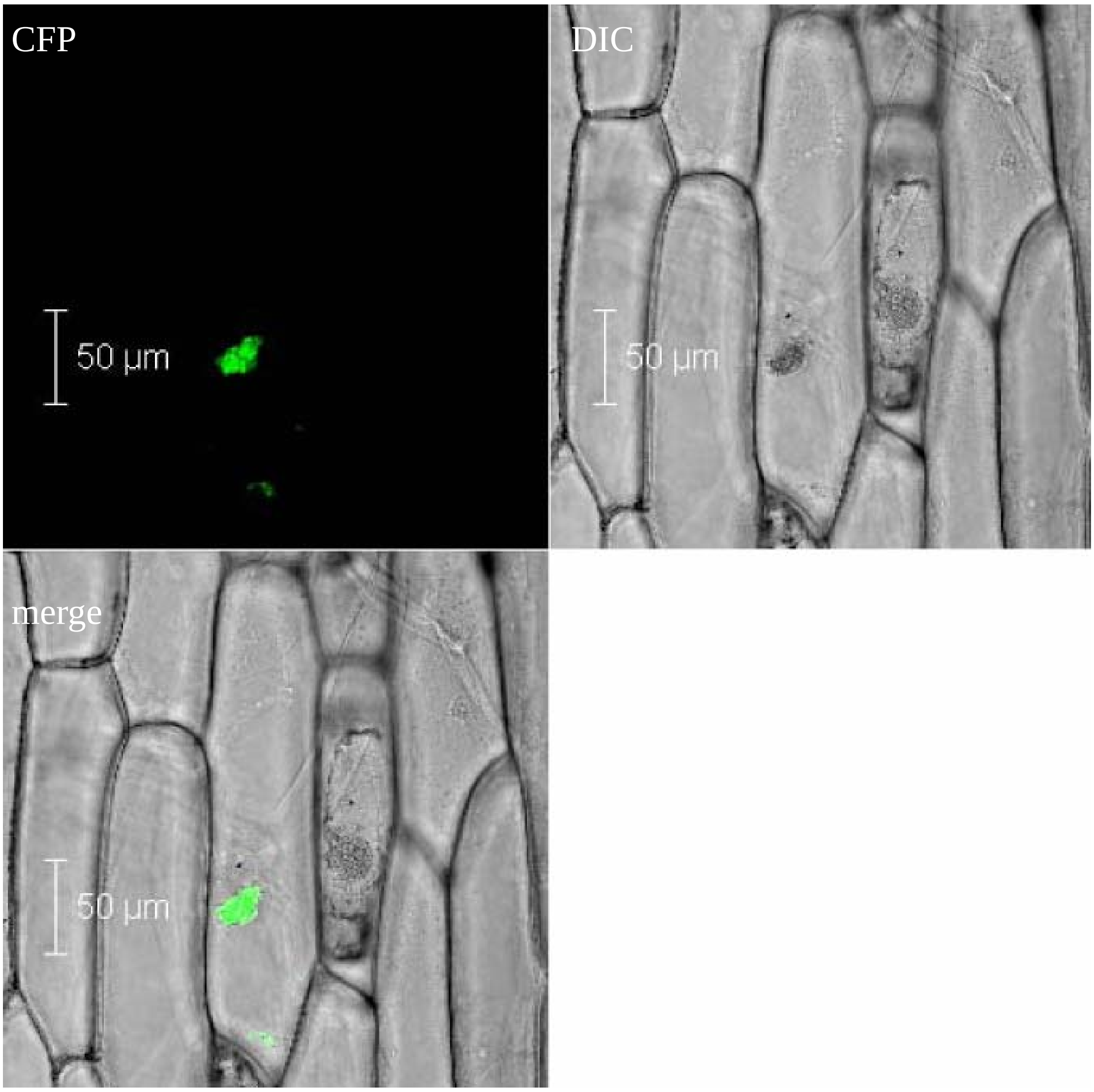
CFP
50 µm
DIC
50 µm
merge
50 µm

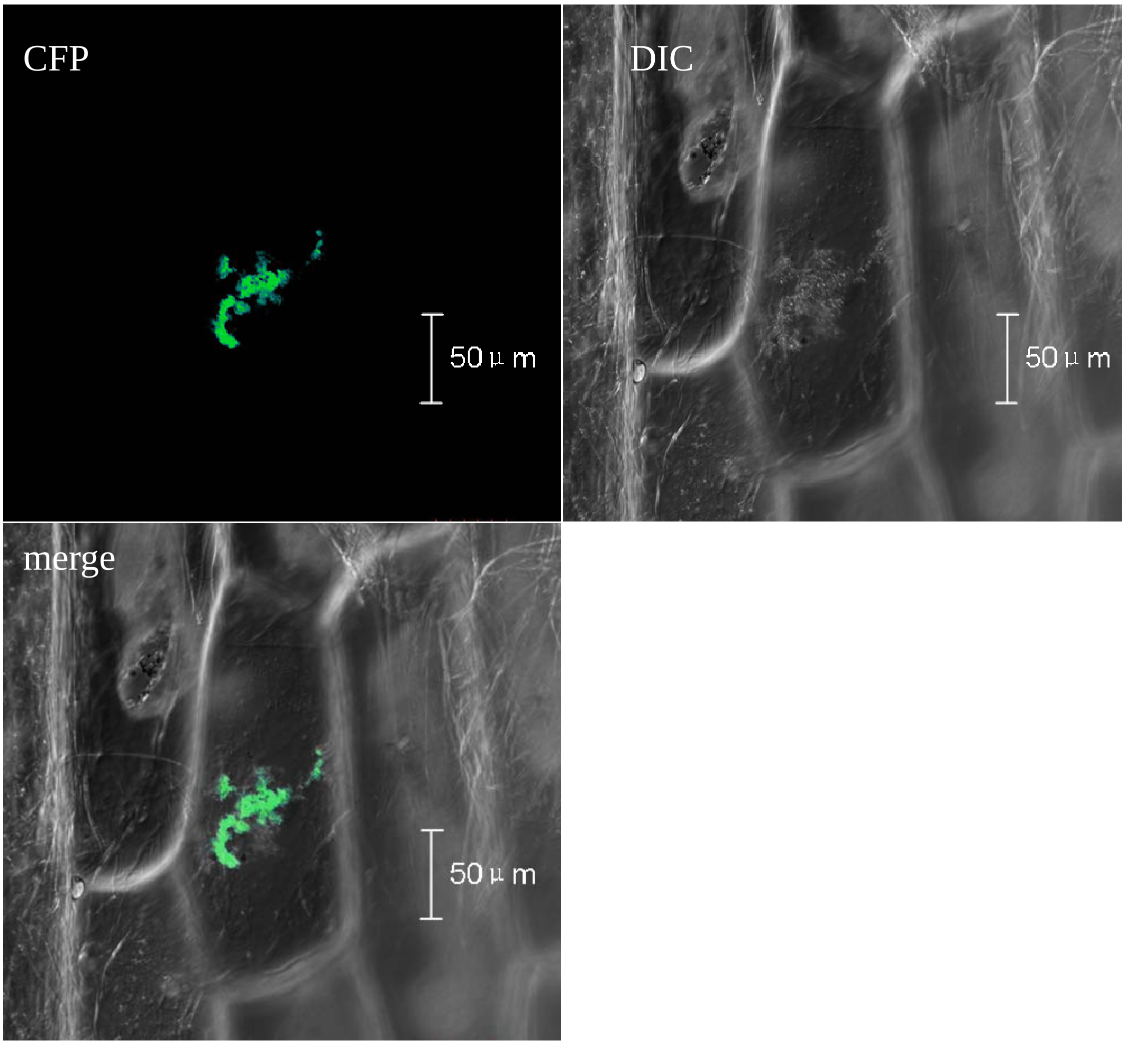

Figure S3. Localization of CFP-Taxilin in onion epidermal cells. Onion epidermal cells were bombarded with CFP-Taxilin. Green represents CFP fluorescence. Differential interference contrast (DIC) was used in light mode.
