# Supplementary material for: Zea mays Taxilin Protein Negatively Regulates Opaque-2 Transcriptional Activity by Causing a Change in Its Sub-Cellular Distribution
Source: PLoS One. 2012 Aug 24;7(8):e43822. doi: 10.1371/journal.pone.0043822 (PMC3427180; doi:10.1371/journal.pone.0043822)
Supplement: Figure S4 — Co-localization of CFP-Taxilin and YFP-O2 in onion epidermal cells. Red represents YFP fluorescence and green represents CFP fluorescence. Differential interference contrast (DIC) was used in light mode. (PDF) [file pone.0043822.s004.pdf]

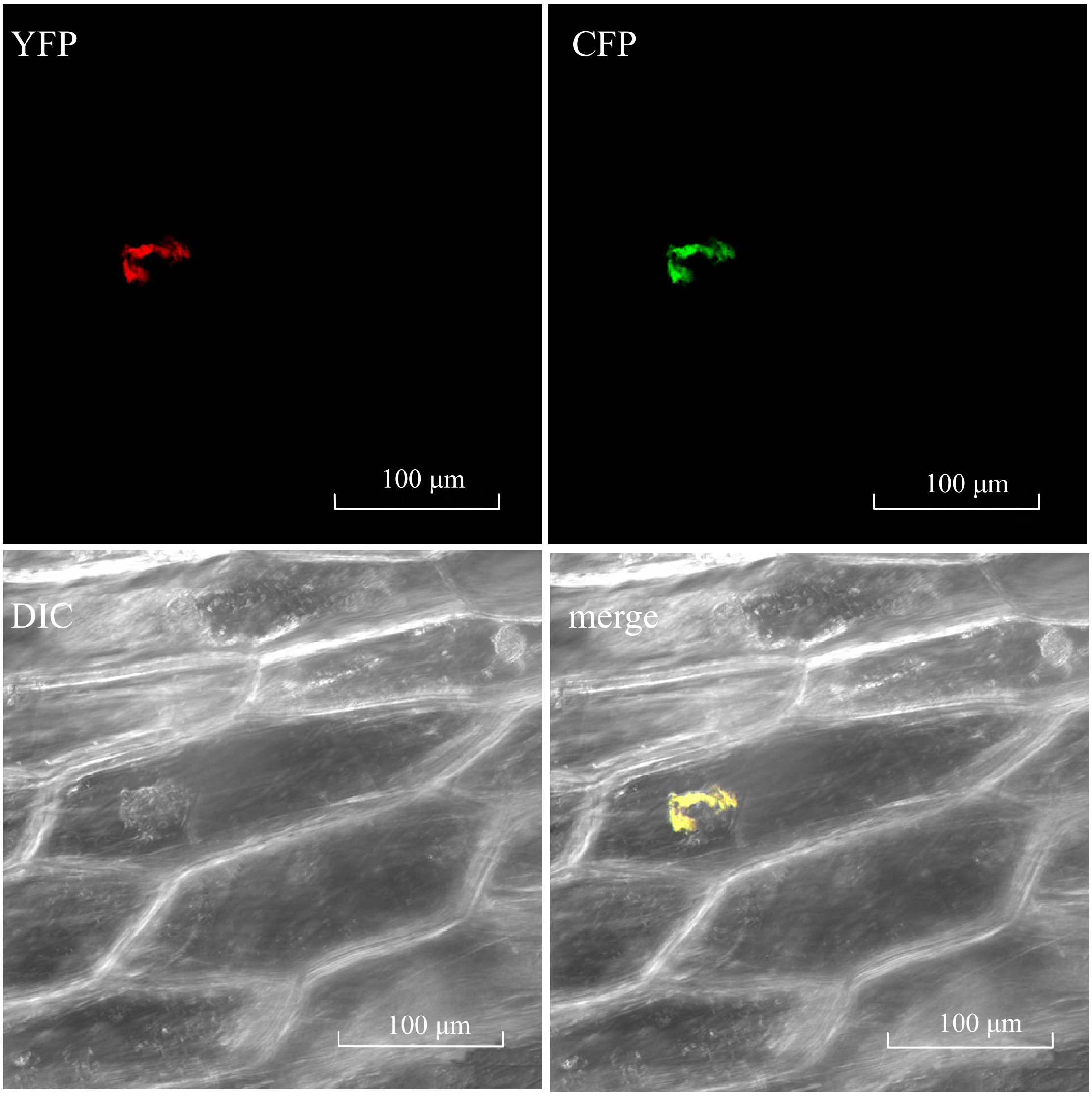
YFP
100 μm
CFP
100 μm
DIC
100 μm
merge
100 μm

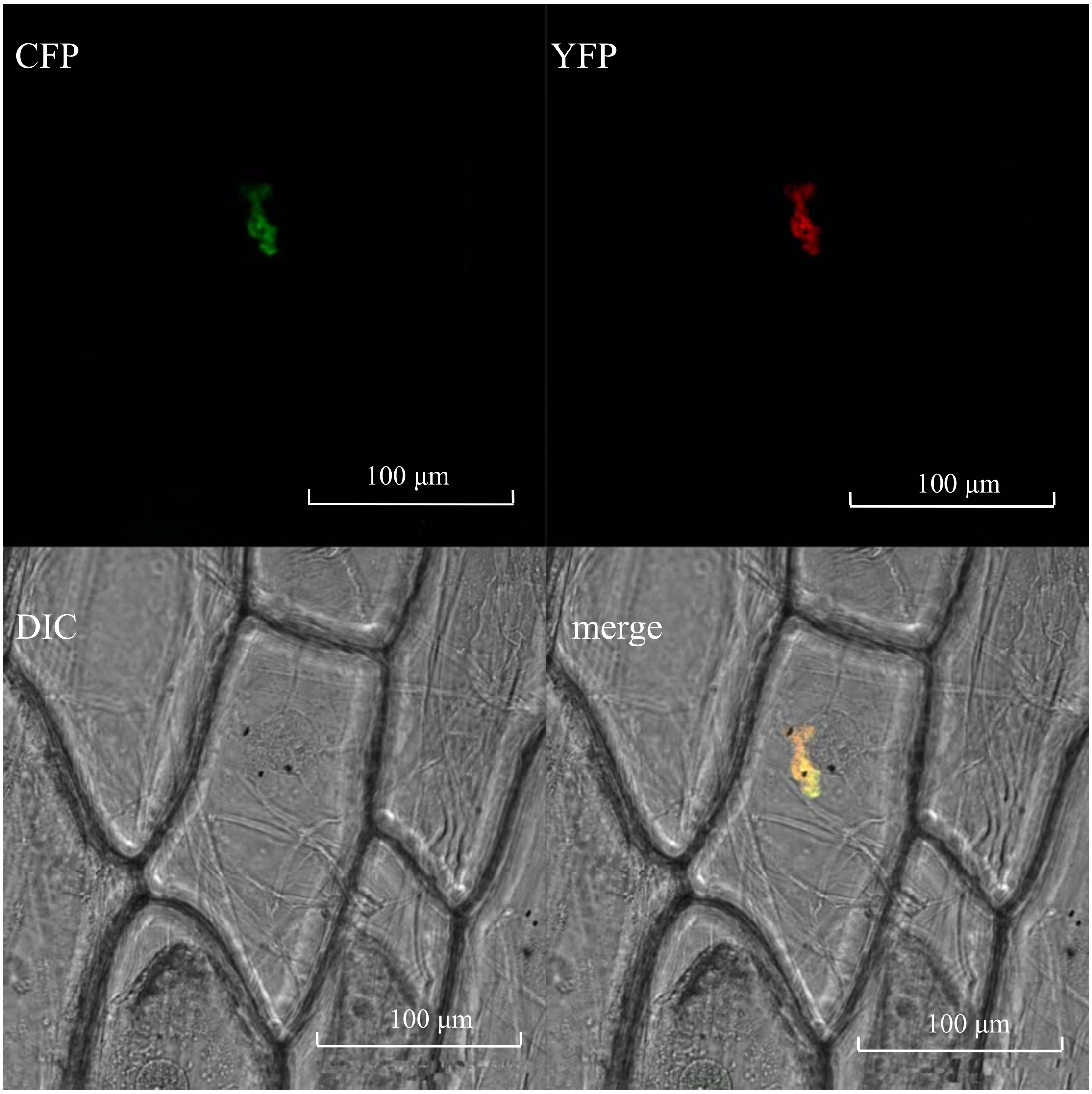
CFP
YFP
100 μm
100 μm
DIC
merge
100 μm
100 μm

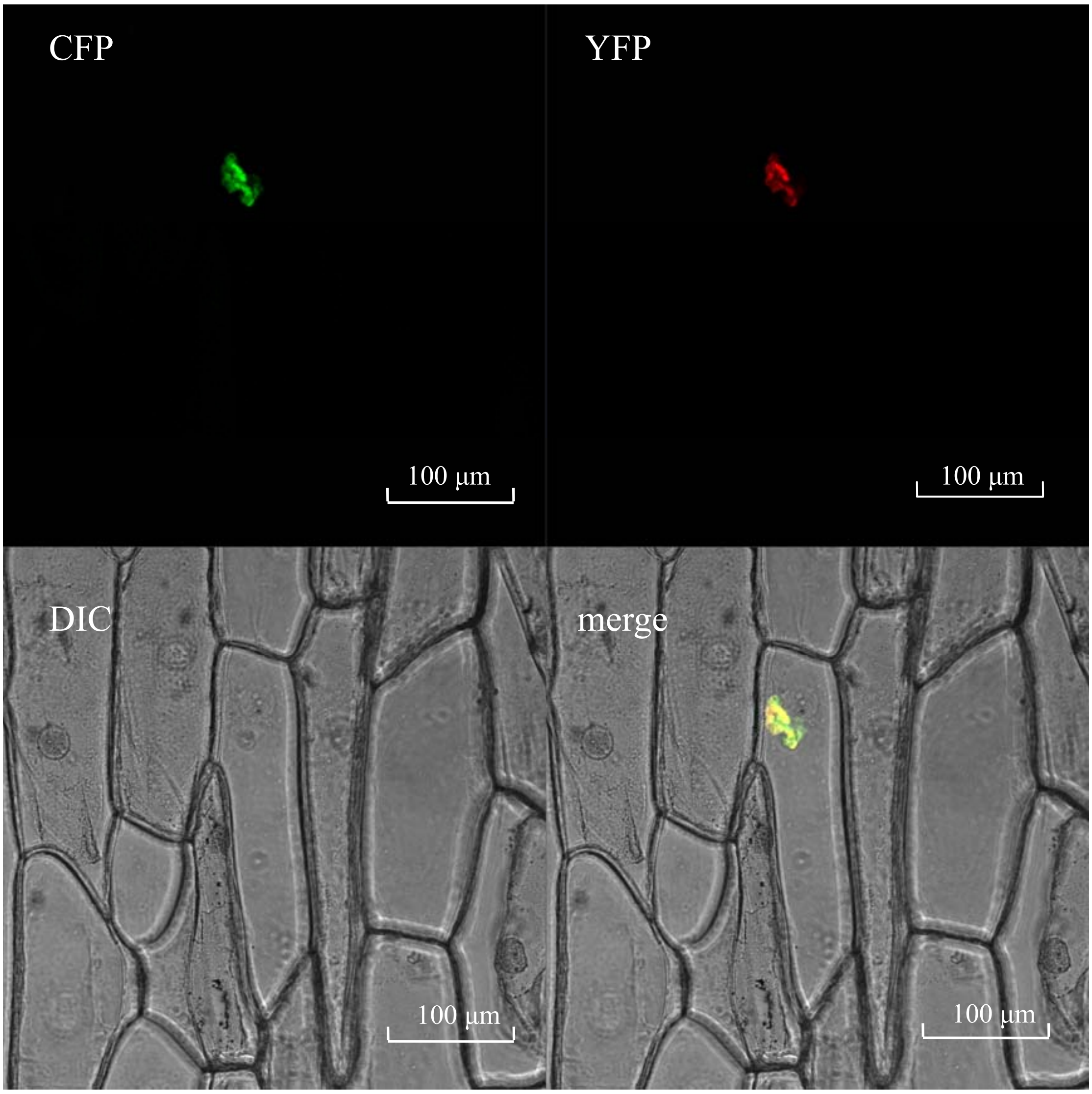


**Figure S4. Co-localization of CFP-Taxilin and YFP-O2 in onion epidermal cells.** Red represents YFP fluorescence and green represents CFP fluorescence. Differential interference contrast (DIC) was used in light mode.
